# Discovery of a Hidden *Trypanosoma cruzi* Spermidine Synthase Binding Site and Inhibitors through *In Silico, In Vitro*, and X-ray Crystallography

**DOI:** 10.1021/acsomega.3c01314

**Published:** 2023-07-12

**Authors:** Ryunosuke Yoshino, Nobuaki Yasuo, Yohsuke Hagiwara, Takashi Ishida, Daniel Ken Inaoka, Yasushi Amano, Yukihiro Tateishi, Kazuki Ohno, Ichiji Namatame, Tatsuya Niimi, Masaya Orita, Kiyoshi Kita, Yutaka Akiyama, Masakazu Sekijima

**Affiliations:** † Transborder Medical Research Center, University of Tsukuba, Tsukuba 305-8577, Japan; ‡ Education Academy of Computational Life Sciences (ACLS), Tokyo Institute of Technology, Yokohama 226-8501, Japan; § Tokyo Tech Academy for Convergence of Materials and Informatics (TAC-MI), Tokyo Institute of Technology, Meguro, Tokyo 152-8550, Japan; ∥ Medicinal Chemistry Research Labs, Drug Discovery Research, Astellas Pharma Inc, Miyukigaoka, Tsukuba 305-8585, Japan; ⊥ School of Computing, Tokyo Institute of Technology, Tokyo 152-8550, Japan; # School of Tropical Medicine and Global Health, Nagasaki University, Sakamoto, Nagasaki 852-8523, Japan; ∇ Department of Biomedical Chemistry, Graduate School of Medicine, The University of Tokyo, Tokyo 113-0033, Japan

## Abstract

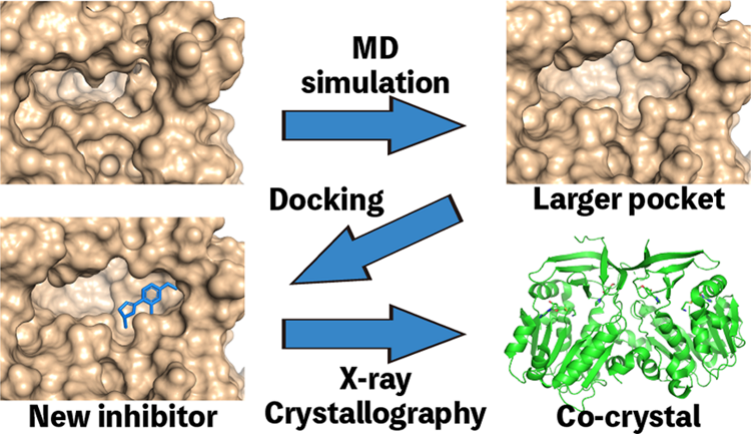

In drug discovery research, the selection of promising binding sites and understanding the binding mode of compounds are crucial fundamental studies. The current understanding of the proteins-ligand binding model extends beyond the simple lock and key model to include the induced-fit model, which alters the conformation to match the shape of the ligand, and the pre-existing equilibrium model, selectively binding structures with high binding affinity from a diverse ensemble of proteins. Although methods for detecting target protein binding sites and virtual screening techniques using docking simulation are well-established, with numerous studies reported, they only consider a very limited number of structures in the diverse ensemble of proteins, as these methods are applied to a single structure. Molecular dynamics (MD) simulation is a method for predicting protein dynamics and can detect potential ensembles of protein binding sites and hidden sites unobservable in a single-point structure. In this study, to demonstrate the utility of virtual screening with protein dynamics, MD simulations were performed on *Trypanosoma cruzi* spermidine synthase to obtain an ensemble of dominant binding sites with a high probability of existence. The structure of the binding site obtained through MD simulation revealed pockets in addition to the active site that was present in the initial structure. Using the obtained binding site structures, virtual screening of 4.8 million compounds by docking simulation, *in vitro* assays, and X-ray analysis was conducted, successfully identifying two hit compounds.

## 1 Introduction

Drug discovery is generally expensive and time-consuming, requiring approximately $2.6 billion and 12–14 years for a drug to reach the market.^1,2^ Computational methods offer a way to reduce these barriers to drug discovery, development, and design. Drug design processes are divided into two types. Ligand-based drug design (LBDD) is based on activity values (such as half-maximal inhibitory concentration, IC_50_), and known compound properties involved in drug binding. Representative methods in LBDD include quantitative structure-activity relationship (QSAR)^3−5^ and machine learning.^6−8^ Alternatively, structure-based drug design (SBDD) bases the design process on a target protein structure. In SBDD, the discovery of the target protein binding site is a fundamental starting point.^9,10^ Typically, the protein binding site is identified by X-ray analysis and the drug is designed or optimized based on information from that analysis. Human immunodeficiency virus 1 (HIV-1) protease inhibitors were developed using SBDD.^11−13^ Thus, binding site information, such as shape and physical properties, is crucial for drug development and optimization.

In general, three protein-ligand binding models are known: lock and key, induced-fit, and pre-existing equilibrium models.^14^ The lock-and-key model, first proposed by Emil Fischer in 1894, is the simplest binding model, in which the ligand fits perfectly into the keyhole of the protein. In contrast, the induced-fit model was proposed by Koshland,^15^ in which the protein pocket changes to match the shape of the ligand. Ligands induce a conformational change at the target site that activates or inactivates the protein by binding to the target site. The pre-existing equilibrium model includes ligand-bound pocket shapes in the ensemble of apo form.^16^ Ligand changes equilibrium to a bound state by selectively binding to the ligand-bound pocket shapes. Drugs that bind to proteins, such as enzymes, are roughly divided into two types: competitive inhibitors and noncompetitive inhibitors. Competitive inhibitors bind to active sites at which the protein catalyzes a reaction, whereas noncompetitive inhibitors bind to nonactive sites, such as allosteric sites. Noncompetitive inhibitors that bind to allosteric sites have several advantages compared with competitive inhibitors that bind to active sites, including low side effects and high affinities.^17^ Thus, the determination of new binding sites such as allosteric sites, is important in drug development studies. However, although all proteins are potentially allosteric,^18^ few cases of allosteric inhibitors have been reported.^19^ Therefore, protein-ligand binding models and inhibition modes are diverse, and clarifying these mechanisms at the molecular level is important in inhibitor discovery and structure optimization.

To detect binding sites for drug design, computational methods to identify binding sites, such as POCKET,^20^ LIGSITE,^21^ CAST,^22^ PASS,^23^ SURFNET,^24^ Q-SiteFinder^25^ and MetaPocket 2.0,^26^ have been reported. These methods estimate the protein binding site from the three-dimensional geometry of the protein, and no ligand is required. Moreover, several studies have adopted machine learning methods, such as the support vector machine (SVM) method, for predicting allosteric sites.^27−29^ In combination with these binding site detection methods, virtual screening methods, such as protein-ligand docking simulations,^30^ have been applied to develop new drugs.^31−33^ DOCK,^34−36^ AutoDock,^37,38^ AutoDock Vina,^39^ GOLD,^40^ and Glide^41,42^ are typical docking simulation software, and there have been many reports of screening with these docking simulations, including protein-ligand complex-derived pharmacophores.^43−60^

Typically, traditional computational methods, such as binding site identification and protein-ligand docking simulations, do not consider protein flexibility because the calculations are for a single point. Thus, these methods provide limited screening as these methods as they rely on lock and key models that treat the system as a rigid body. It is essential to consider the dynamic features of proteins in order to perform virtual screening that yields a greater variety of compounds.

MD simulations account for protein flexibility using Newtonian principles. This method can predict protein-ligand induced-fit and ensembles of pre-existing equilibrium models by predicting the dynamics of proteins. Moreover, Ma *et al*. reported a computational method for predicting allosteric sites from residue-residue interaction patterns.^61^ In that study, conformational ensembles of a target protein generated by MD simulations for site prediction were applied. Thus, MD simulations can be used for identifying new binding modes and pockets that traditional computational methods cannot.

In this study, to demonstrate the effectiveness of virtual screening for protein flexibility, we performed virtual screening using docking simulation and MD simulations for the binding site of *Trypanosoma cruzi* spermidine synthase (TcSpdSyn)^62−67^ that cannot be detected by single-point methods. We then performed *in vitro* assays to determine the inhibition activities of compounds identified by the docking simulations and conducted subsequent X-ray crystallographic studies of the active compounds. Finally, we carried out fragment molecular orbital (FMO) calculations to analyze important interactions between TcSpdSyn and the active compounds.

## 2 Materials and Methods

### 2.1 Computational Methods

The structure of TcSpdSyn (PDB ID: 3BWC), as the docking target, was obtained from the Protein Data Bank. The hydrogen assignment to the protein, water removal, and conformation optimization of the complex were accomplished in Maestro using the OPLS2005 force field.^68^ And carboxyl group of S-adenosylmethionine (SAM) which is included in the structure was deleted to correct SAM to Decarboxylated S-adenosylmethionine (dcSAM) as a cofactor. The MD simulation system was prepared using Desmond ver. 3.5 with the default settings. The temperature and pressure of the system were set to 300 K and 1 atm, respectively. The time step and structure sampling interval were set to 2 fs and 1 ps, respectively. We performed the simulation five times under the NPT ensemble for 20 ns. Next, we merged all trajectories from the MD simulation and performed structure clustering based on the amino acid residues at active site, which are shown in the Table S1, using average linkage in AMBER.^69^ After clustering, site volume and druggability of the active center were evaluated by SiteMap.^70^

Docking simulations were performed at the active site of the prepared structure in the absence of the natural substrate putrescine. For the docking simulation, a 20 × 20 × 20 Å^3^ grid box was generated, thereby maintaining the TcSpdSyn active site. dcSAM, as a cofactor, was not deleted. We used Glide in standard precision (SP) mode^41,42^ for our docking simulations of approximately 4,800,000 drug-like compounds in the Namiki Sho-ji Co., Ltd., library and the Astellas Pharma Inc. in-house compound library that satisfy Lipinski’s rule of five.^71^ All calculations were performed on an HP Proliant SL390s G7 server with an Intel Xeon X5670 2.93 GHz core and five nodes on the TSUBAME2.5 supercomputer at the Tokyo Institute of Technology. The X-ray crystallography structures of TcSpdSyn with compounds 1 and 2 were hydrogenated in Maestro using the OPLS2005 force field. FMO calculation input files were generated using FMOutil Version 2.1, and calculations were performed for the TcSpdSyn complexes with 1 and 2 using GAMESS^72^ at the MP2/6-31G level. Interaction energy analysis was performed using the analytical tool Facio,^73^ which is based on pair interaction energy decomposition analysis, as proposed by Fedorov and Kitaura.^74^

### 2.2 *In Vitro* Assay

The protocol for the TcSpdSyn inhibition assay has been described previously.^75^ The assay was performed using an enzyme-coupled assay incorporating spermidine/spermine N(1) -acetyltransferase 1 (SSAT1). 7-Diethylamino-3-(4’-maleimidylphenyl) -4-methylcoumarin (cat. D-346, Thermo Fisher Scientific) was used to measure coenzyme A produced from the SSAT1 reaction. Briefly, a reaction mixture of 4-(2-hydroxyethyl)-1-piperazineethanesulfonic acid (HEPES) buffer (50 mM, pH 7.5) containing ethylenediaminetetraacetic acid (EDTA, 10 μM), 0.01% Tween 20, TcSpdSyn (14.7 nM), dcSAM (50 μM), putrescine (50 μM), acetyl coenzyme A (15 μM), and SSAT1 (0.83 nM) in the presence or absence of 1 or 2 was incubated at room temperature for 30 min. The concentrations of putrescine and dcSAM were determined using their Km values (data not shown). The fluorescence signals were detected using a Paradigm plate reader (Molecular Devices) with excitation at 405 nm and emission at 530 nm. IC_50_ values were calculated from dose-response curves in which each of eight data points represents the average of four measurements (Figure S2). Compound 2 was used as the hydrochloride salt. These compounds were dissolved in dimethyl sulfoxide (DMSO), the final concentration of which in the assays was as high as 1.3%.

### 2.3 X-ray Crystallography Analysis

The protocol for X-ray crystallography has been described previously.^75^ Briefly, co-crystals of TcSpdSyn complexed with dcSAM and compound 1 were obtained using the sitting-drop vapor diffusion method. Prior to crystallization, TcSpdSyn (15 mg/mL) was mixed with dcSAM and compound 1 at final concentrations of 2 and 5 mM, respectively. A reservoir solution consisting of bis-Tris (100 mM, pH 5.5-6.5), ammonium sulfate (200 mM), and 10-15% (w/v) PEG4000 was prepared. The precipitated crystals were transferred into a mother liquor containing 20% (v/v) glycerol as a cryoprotectant, which was then flash frozen in liquid nitrogen. X-ray diffraction data were collected at the Photon Factory (Tsukuba, Japan) AR-NE3A beamline using a robotic sample changer and an automated data collection system.^76,77^ The structure was resolved by molecular replacement using Phaser.^78^ The apo-structure of TcSpdSyn (PDB ID: 3BWB) was used as a reference model. After structural refinement using REFMAC,^79^ dcSAM and compound 1 were clearly observed in the electron density maps and fitted to the maps using AFITT (OpenEye Scientific). The final structures were deposited in the Protein Data Bank (PDB IDs: 5Y4P and 5Y4Q).

## 3 Results

### 3.1 Discovering of Hidden Binding Site by Molecular Dynamics

**Figure 1:**
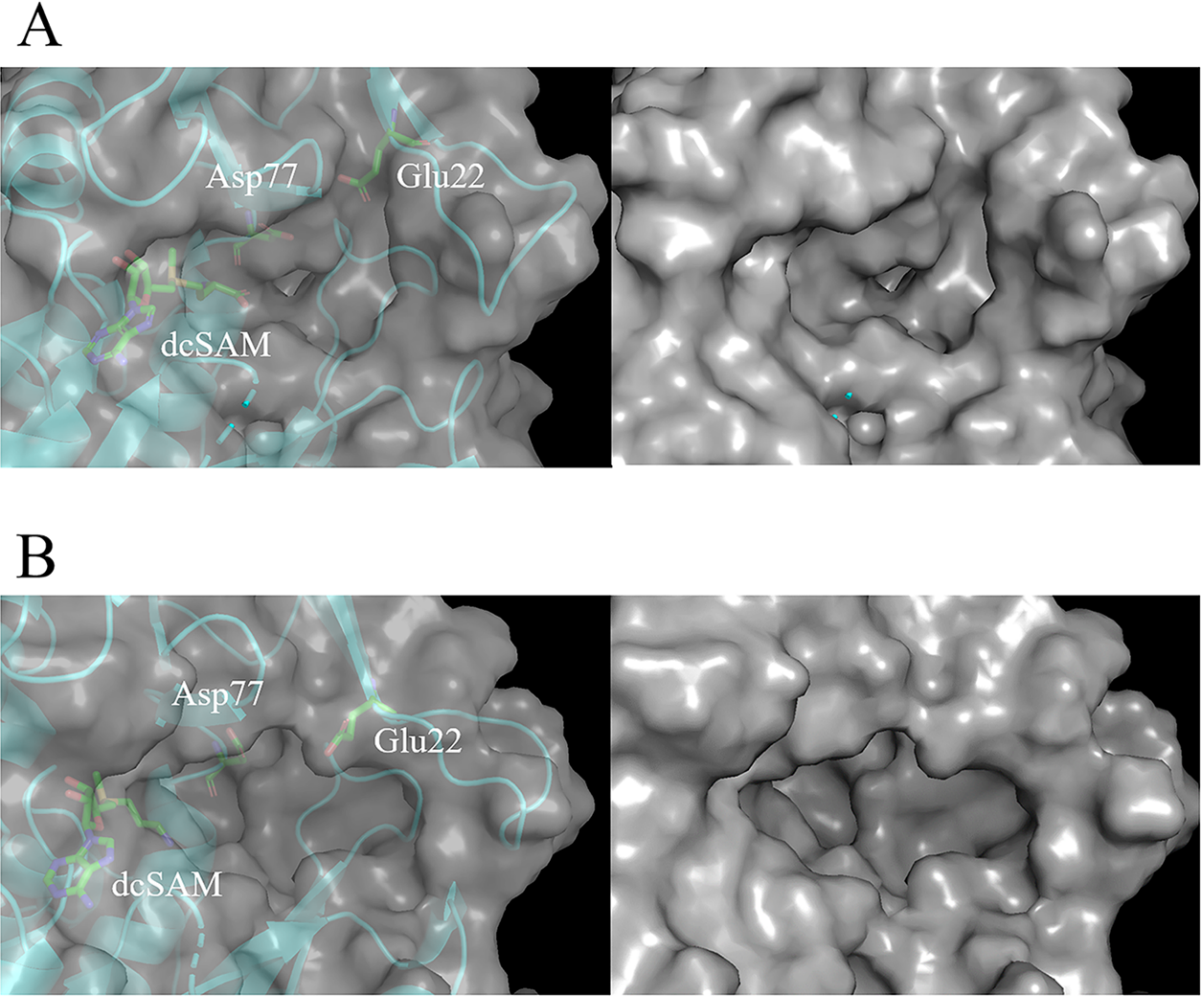
TcSpdSyn target site in the X-ray and clustering structures. (A) X-ray structure (volume: 193 Å^3^, D-score: 0.56), (B) clustering structure 1 (volume: 496 Å^3^, D-score: 1.12, population: 0.178).

To predict TcSpdSyn binding sites, we performed MD simulations and structure clustering for virtual screening. Figure S1 shows the root-mean-square deviations (RMSD) of TcSpdSyn α-carbon atoms, side chains, and heavy atoms during a 20 ns MD simulation. Next, we conducted structure clustering to extract representative structures from the trajectory. Figure 1 shows the active site of TcSpdSyn in the X-ray structure and clustering structures.

The active site volume of the X-ray structure was 193 Å^3^. However, the active site volumes of the clustering structures were 496 Å^3^. In clustering structure 1 (Figure 1B), a new cavity, which was not identified in the X-ray structure, was discovered around Glu22. We also evaluated these binding sites by the D-score of SiteMap (Schrod̈inger inc.) The D-scores indicating the druggability of the clustering structures were higher than that of the X-ray structure (X-ray D-score: 0.56, clustering structure 1 D-score: 1.12). Target sites with D-scores higher than 0.98 are highly druggable.^70,80^ These results suggest that the target site of TcSpdSyn is flexible and has a structure with higher druggability potential. It is possible that compounds not found when using the X-ray structure could be evaluated by using the predicted structure. We suggested that molecules that inhibit structural change can bind to the new site. We defined the new site as a hidden binding site and then performed docking simulations for the hidden binding sites in the clustering structures. We also performed docking simulations for the active site in the X-ray structure to compare docking simulation results between hidden binding sites and the active site in the X-ray structure.

### 3.2 *In Silico* Screening by Docking Simulation

**Figure 2:**
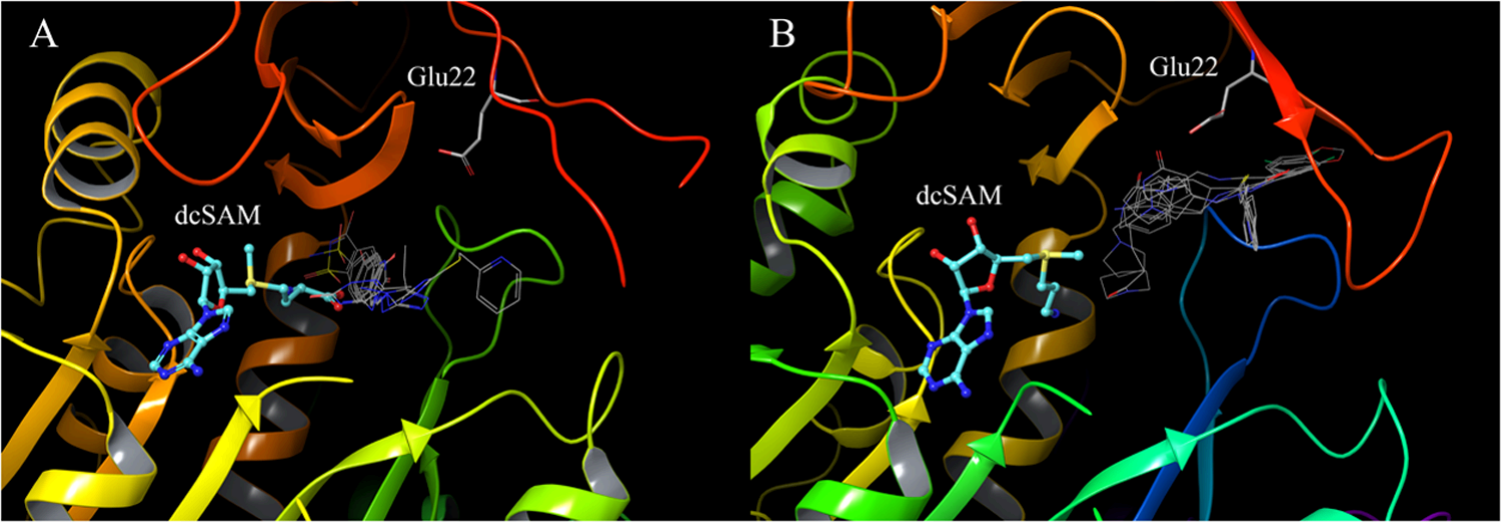
Comparison of docking poses of the top five compounds with high docking scores at each binding site. (A) docking pose of the X-ray structure, (B) docking pose of the clustering structure. The stick model shows Glu22 and dcSAM, and the line model shows docking results.

To obtain drug candidates from our combined library of 4,800,000 drug-like compounds, we conducted docking simulations for the TcSpdSyn hidden binding site, as predicted by MD simulations and the active site in the X-ray structure, using Glide in the SP mode. Figure 2 shows the docking poses of the top five compounds with high docking scores at each binding site.

**Figure 3:**
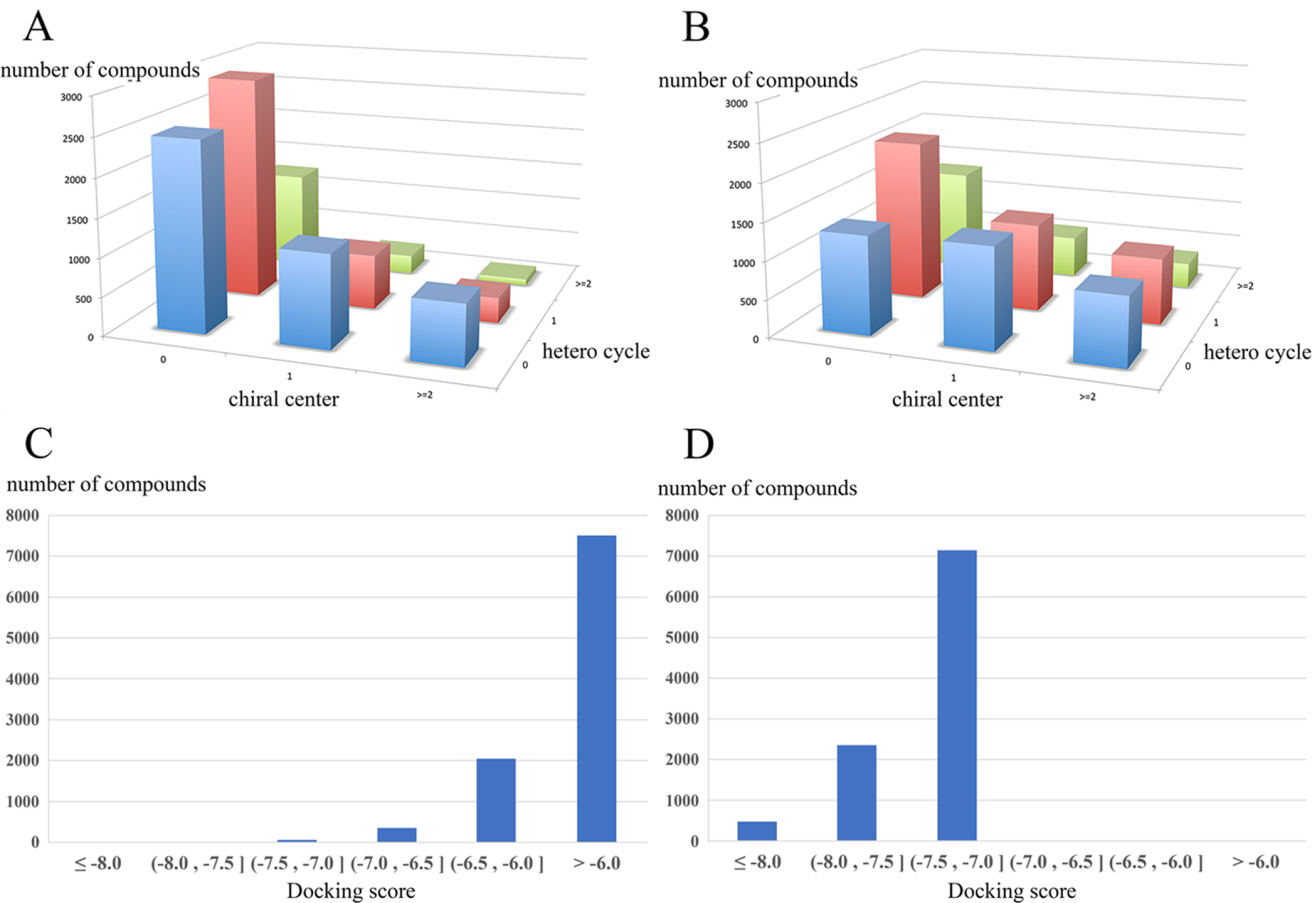
Diversity of docking results and score histograms for the top 10,000 compounds. (A) chiral center and hetero cycle number of X-ray structure docking results, (B) chiral center and hetero cycle number of clustering structure 1 docking results, (C) docking score histogram of X-ray structure docking results, (D) docking score histogram of clustering structure 1 docking results.

The docking results of the X-ray structure show that these compounds bind to the TcSpdSyn active center, which is adjacent to dcSAM. In contrast, the docking poses in the clustering structures cover a wide range of hidden binding sites. Figure 3 shows the diversity of the top 10,000 compounds with high docking scores in each docking result. In the X-ray structure (Figure 3A), many compounds lacking a heterocycle or chiral center are favored. In contrast, more compounds containing a heterocycle and/or chiral center are favored with clustering structure 1 (Figure 3B). Figures 3C,D also show docking score histograms for the top 10,000 compounds. Histograms of X-ray structures have the most results above −6.0, whereas histograms of clustering structures have the most compounds between −7.5 and −7.0. Overall, our docking simulations identified a variety of compounds after performing MD simulations and structure clustering.

### 3.3 *In Vitro* Assay and X-ray Crystallography Analysis

We ran docking simulations targeted to the ”virtual” hidden binding site found in the MD simulations. Next, we selected 191 compounds in order of highest docking score for the hidden binding site and performed *in vitro* enzyme assay to validate their IC_50_ concentration values. The results showed that two compounds exhibited inhibitory activity (Table 1).

**Table 1:**
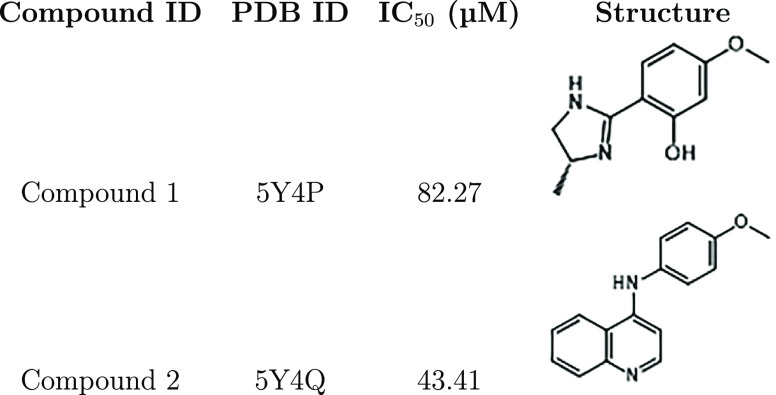
Summary of TcSpdSyn Inhibition by Compounds 1 and 2^a^

a PDB IDs for the co-crystallized enzyme-inhibitor complexes, IC_50_ values, and the molecular structures of the inhibitors are shown.

**Figure 4:**
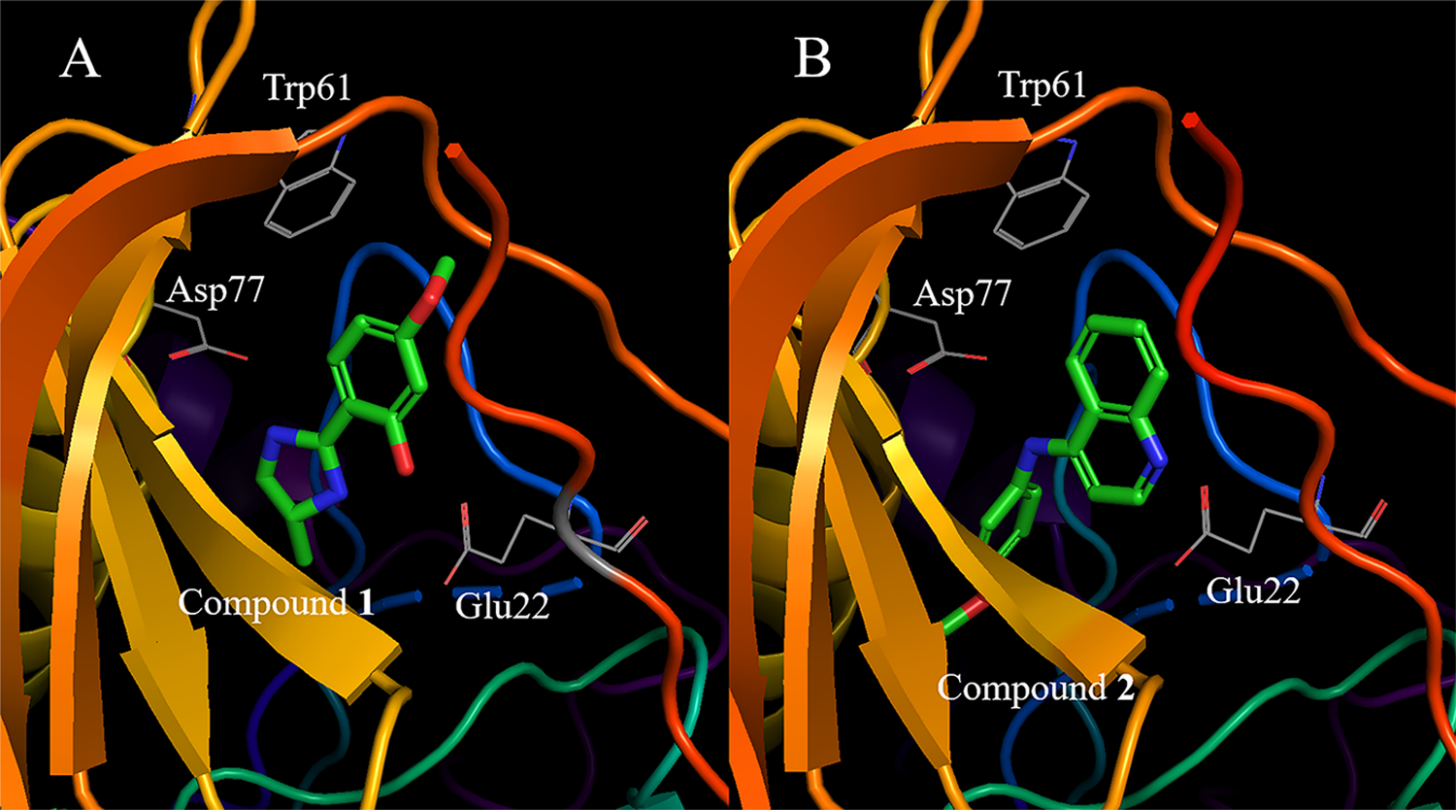
Binding site of each compound confirmed by X-ray analysis. (A) TcSpdSyn with compound 1 (PDB ID: 5Y4P), (B) TcSpdSyn with compound 2 (PDB ID: 5Y4Q).

To examine the binding sites used by these two active compounds, we conducted X-ray crystallographic analyses to observe the structures of the TcSpdSyn complex with the two top-ranked compounds (compounds 1 and 2) in the hidden binding pocket (Figure 4), as predicted by the MD simulations.

**Figure 5:**
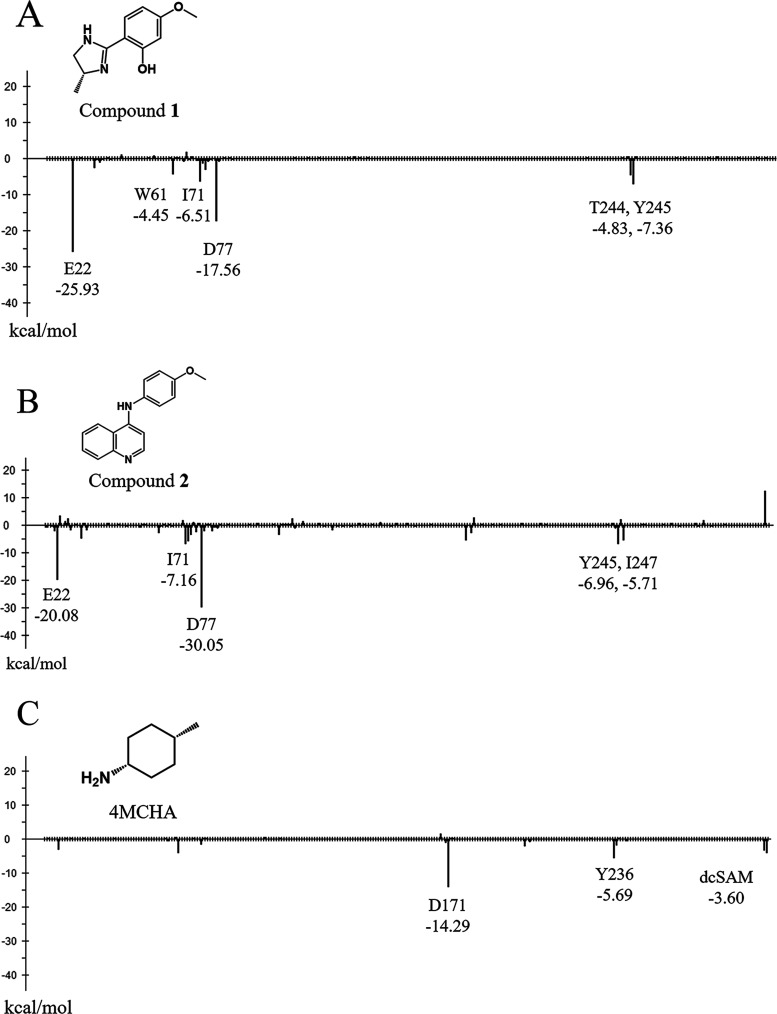
Interaction energy analysis of each X-ray structure. (A) interaction energy of compound 1, (B) interaction energy of compound 2. (C) interaction energy of cis-4-methylcyclohexanamine (4MCHA, PDB ID: 2PT9). The *y*-axis represents the interaction energy (kcal/mol) between the ligand and each amino acid residue, and the x-axis represents the amino acid residue number.

These data show that compound 1 interacts with Glu22 and Asp77 through hydrogen bonding (Figure 4A). Compound 2 interacts with Glu22 and Asp77, similar to 1, and the lone pair of the quinoline nitrogen atom in 2 is proximal to the carboxylate group of Glu22. Thus, these results suggest that Glu22 is in a neutral state when interacting with the lone pair of quinoline. Next, we conducted an interaction energy analysis for each X-ray structure using FMO calculations. Figure 5A shows the results of the interaction energy analysis of the TcSpdSyn-1 complex.

Compound 1 interacts with Glu22 and Asp77 (interaction energy values: −25.93 and −17.56 kcal/mol, respectively) through two hydrogen bonds. Therefore, these interactions would appear to be important for binding to the site. Some other interactions were also found: Trp61, Ile71, Thr244, and Tyr245 interacted with compound 1 with interaction energies of −4.45, −6.51, −4.83, and −7.36 kcal/mol, respectively.

**Figure 6:**
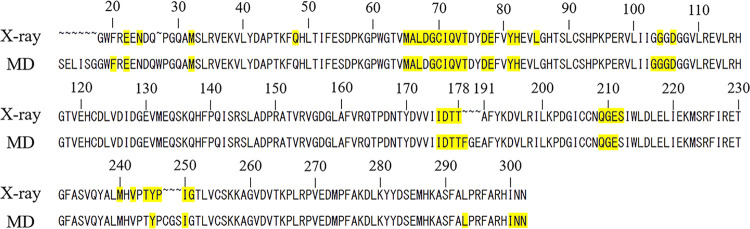
Amino acid residue sequence of the binding sites. X-ray: sequence of the TcSpdSyn-1 complex (PDB ID: 5Y4P), MD: sequence of the clustering structure identified from the MD simulations. The residues were determined using yellow at the binding site. A binding site is defined as the residues within 10 Å of an atom defined by LIGSITEcsc.

Figure 5B shows the results of the interaction energy analysis of the TcSpdSyn-2 complex. Compound 2 interacted with Glu22 and Asp77 (interaction energy values: −20.08 and −30.05 kcal/mol, respectively) through two hydrogen bonds in the same manner as 1. In particular, Asp77 interacted with compound 2 in a neutral state. Moreover, some weak interactions, such as with Ile71 Tyr245 and Ile247, were confirmed, with interaction energy values of −7.16, −6.96, and −5.71 kcal/mol, respectively. Figure 5C shows the results of the interaction energy analysis of the TcSpdSyn-cis-4-methylcyclohexanamine (4MCHA) complex (PDB ID: 2PT9).^81^ 4MCHA has been reported as a known inhibitor and binds to the TcSpdSyn active site.^81^ This inhibitor interacted with Asp171 (interaction energy: −14.29 kcal/mol). Furthermore, 4MCHA also interacted with dcSAM, which is a cofactor of SpdSyn. These results suggested that compound 1 and 2 show interaction patterns different from 4MCHA. Figure 6 shows the amino acid sequence of the binding sites defined by LIGSITEcsc.^82^

Upon examination of the X-ray structure of TcSpdSyn with compound 1 at the binding site, the amino acid sequence overlap with the clustering structure was 72.2%. Glu22, which interacts with 1, is a feature of the sequence of the clustering structure binding site. Therefore, the MD simulations predicted the new binding site of TcSpdSyn and the amino acid residues that contribute a significant interaction at the binding site.

## 4 Discussion

We employed a molecular simulation approach, conducting MD simulations to predict novel TcSpdSyn binding sites. These simulations revealed a new binding site shape not apparent in the X-ray structure. The MD simulations-predicted binding site exhibits a higher D-score and a larger volume compared to the X-ray structure. This binding site emerges due to structural changes in the protein. one of the potential ensembles TcSpdSyn can adopt when in equilibrium in an aqueous solution. Such a protein structure cannot be discovered by observing a single limited state like a crystal structure.

To identify seed compounds for potential TcSpdSyn inhibitors, we performed docking simulations using the TcSpdSyn X-ray structure and clustering structures. These simulations identified active compounds from approximately 4.8 million drug-like compounds. Based on the X-ray structure, drug candidates without a heterocycle and chiral center were considered. Conversely, drug candidates containing a heterocycle and/or chiral center were considered for clustering structure 1, as predicted by the MD simulations. Combining docking and MD simulations enables the evaluation of a diverse range of compounds. The histogram of docking scores reveals that clustering structure 1 exhibits higher affinity than the docking results from the X-ray structure. Generally, docking scores are influenced by factors such as the number of hydrogen bonds and protein interaction surfaces. Consequently, clustering structure 1, with its larger site volume, is advantageous in docking simulations, as hydrogen bonding and interacting surfaces are more easily attainable than in the binding site of the X-ray structure.

**Figure 7:**
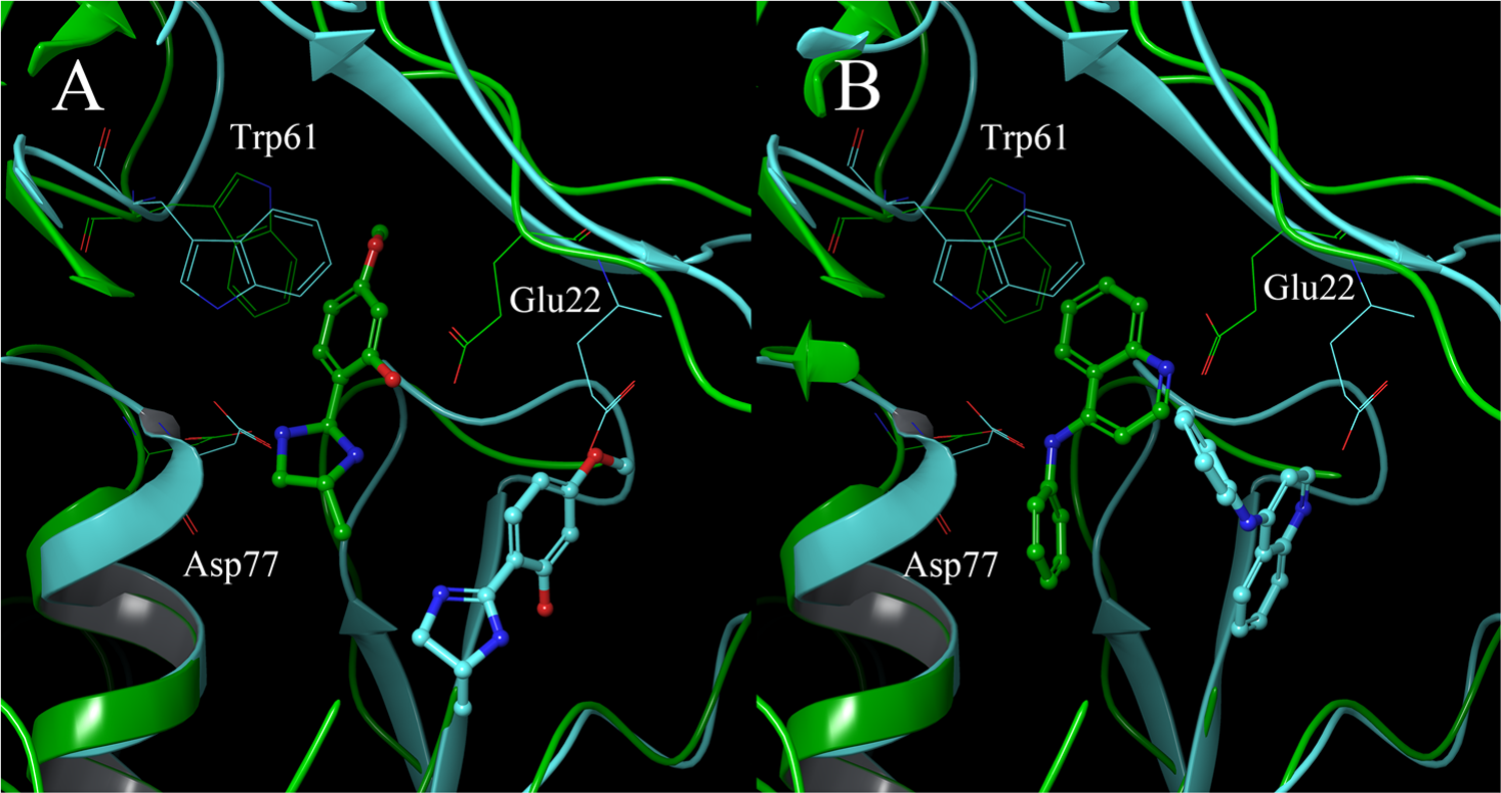
Comparison of compound conformations in docking and X-ray structures. Blue and green represent docking results and X-ray structures, respectively. (A) comparison of compound 1, (B) comparison of compound 2.

To assess their IC_50_ values, drug candidates from the docking results were screened in TcSpdSyn inhibition assays. As a result, TcSpdSyn IC_50_ values for two compounds were determined, with compounds 1 and 2 inhibiting TcSpdSyn at IC_50_ values of 82.27 and 43.41 μM, respectively. To verify the binding mode, we determined the X-ray structure of the TcSpdSyn-ligand complexes. The crystal structures showed that compounds 1 and 2 bind to the hidden binding site, as predicted by the simulations, and interact with Glu22 and Asp77 through hydrogen bonds. These hydrogen bonds are absent in the TcSpdSyn active site structure where putrescine is bound. Comparing the structures of compounds 1 and 2, both are para-substituted anisoles with nitrogen-rich heterocycle para-substituents. However, the X-ray structures display opposite orientations for compounds 1 and 2 at the new site. Compound 1 interacts with Glu22 through the hydroxy group at the meta-position of anisoles, while compound 2 interacts with Glu22 via a secondary amine at para-substituent. Consequently, the two compounds exhibit different poses despite sharing a common structure. Figure 7 compares the binding poses of the docking results and X-ray structures. The pose of the docking result is situated near Glu22, while the X-ray structure is also proximate to Trp61 and Glu77, achieving interaction. We have also confirmed that the compounds can approach Trp61 and Glu77 from 50 ns as the results of the pose-refinement MD simulations from the docking poses (Figure S3). Several previous studies have also performed the validity of the pose-refinement MD simulations from the docking poses, with the potential to bind to the target protein to reproduce the induced fit.^83,84^

This likely represents a conformational change and an induced fit when TcSpdSyn accommodates the compound. Such changes could be predicted by performing MD simulations on protein-candidate complex structure models obtained through docking simulations. However, during virtual screening, it is impractical to perform MD simulations for all candidate compound docking poses. Thus, the optimal strategy is to select a few candidate compounds with the highest docking scores and apply MD simulations to predict the effects of these compounds on the protein structure. The target site for virtual screening, when combined with MD simulation, is situated adjacent to the active site. Known inhibitors binding to the TcSpdSyn active site, such as 4MCHA, have been reported. We also have reported inhibitors binding to the TcSpdSyn active site of.^85^

In conclusion, our study demonstrates the potential of combining molecular dynamics (MD) simulations with docking simulations to identify novel binding sites and design new active compounds. By using this integrated approach, we successfully predicted a previously undiscovered binding site on *Trypanosoma cruzi* spermidine synthase (TcSpdSyn) and identified two active compounds with inhibitory activity. The determination of two co-crystal structures with these active compounds further confirmed the concept of the strategy. The virtual screening method considering protein dynamics allows for the exploration of various design strategies, enhancing the drug discovery process. Our findings highlight the value of employing a combination of MD and docking simulations for computational drug discovery, especially for challenging targets such as TcSpdSyn.

## Data Availability

The 3D structures of the proteins have been deposited in the Protein Data Bank (PDBIDs are 5Y4P and 5Y4Q).

## Supporting Information Available

Reference of amino acid residues for structure clustering (Table S1); Crystallography data for the TcSpdSyn-1 and TcSpdSyn-2 complexes (Table S2); SMILES of inactive compounds (Table S3); Root-mean-square deviations (RMSD) in the MD simulations (Figure S1); fitting curves for the calculation of the IC_50_ values in Table 1 (Figure S2); Prediction of structural changes in docking poses by MD simulation (Figure S3) (PDF)
