# Supplementary material for: Discovery of a Hidden Trypanosoma cruzi Spermidine Synthase Binding Site and Inhibitors through In Silico, In Vitro, and X-ray Crystallography
Source: ACS Omega. 2023 Jul 12;8(29):25850–60. doi: 10.1021/acsomega.3c01314 (PMC10373461; doi:10.1021/acsomega.3c01314)
Supplement: Supplementary file 1 — ao3c01314_si_001.pdf [file ao3c01314_si_001.pdf]

SUPPORTING INFORMATION FOR

Discovery of a Hidden *Trypanosoma cruzi* Spermidine Synthase Binding Site and Inhibitors through *In Silico, In Vitro*, and X-ray Crystallography

Ryunosuke Yoshino,[†,▽] Nobuaki Yasuo,[‡] Yohsuke Hagiwara,[¶,▽] Takashi Ishida,[§]

Daniel Ken Inaoka,[//,⊥] Yasushi Amano,[¶] Yukihiro Tateishi,[¶] Kazuki Ohno,[¶,▽,1] Ichiji Namatame,[¶] Tatsuya Niimi,[¶,2] Masaya Orita,[¶,3] Kiyoshi Kita,[//,⊥] Yutaka Akiyama,[§,▽] and Masakazu Sekijima[*,§,▽]

[†] Transborder Medical Research Center, University of Tsukuba, Tsukuba 305-8577, Japan

[‡] Tokyo Tech Academy for Convergence of Materials and Informatics (TAC-MI), Tokyo Institute of Technology, Meguro, Tokyo 152-8550, Japan

[¶] Medicinal Chemistry Research Labs, Drug Discovery Research, Astellas Pharma Inc, Miyukigaoka, Tsukuba 305-8585 Japan

[§] School of Computing, Tokyo Institute of Technology, Tokyo 152-8550, Japan

[//] School of Tropical Medicine and Global Health, Nagasaki University, Sakamoto, Nagasaki 852-8523, Japan

[⊥] Department of Biomedical Chemistry, Graduate School of Medicine, The University of

Tokyo, Tokyo 113-0033, Japan

[▽] Education Academy of Computational Life Sciences (ACLS), Tokyo Institute of Technology, Yokohama 226-8501 Japan

E-mail: sekijima@c.titech.ac.jp

---

[1] Present affiliation: Modulus Discovery, Inc.

[2] Present affiliation: PeptiDream Inc.

[3] Present affiliation: Axcelead Drug Discovery Partners,Inc.

Table S1. Reference of amino acid residues for structure clustering

Gln48	N, CA, C, O, CB, CG, CD, OE1, NE2
Leu50	N, CA, C, O, CB, CG, CD1, CD2
Met65	N, CA, C, O, CB, CG, SD, CE
Ala66	N, CA, C, O, CB
Leu67	N, CA, C, O, CB, CG, CD1, CD2
Asp68	N, CA, C, O, CB, CG, OD1, OD2
Cys70	CA, C, O, CB, SG
Ile71	N, CA, C, O, CB, CG1, CG2, CD1
Gln72	N, CA, C, O, CB, CG, CD, OE1, NE2
Val73	N, CA, C, O, CB, CG1, CG2
Thr74	N, CA, C, O, CB, OG1, CG2
Glu78	N, CA, C, O, CB, CG, CD, OE1, OE2
Val80	N, CA, C, O, CB, CG1, CG2
Tyr81	N, CA, C, O, CB, CG, CD1, CD2, CE1, CE2, CZ, OH
His82	N, CA, C, O, CB, CG, ND1, CD2, CE1, NE2
Val84	N, CA, C, O, CB, CG1, CG2
Leu85	N, CA, C, O, CB, CG, CD1, CD2
Gly86	N, CA, C, O
Ile101	N, CA, C, O, CB, CG1, CG2, CD1
Ile102	N, CA, C, O, CB, CG1, CG2, CD1
Gly103	N, CA, C, O
Gly104	N, CA, C, O
Gly105	N, CA, C, O
Asp106	N, CA, C, O, CB, CG, OD1, OD2
Gly107	N, CA, C, O
Gly108	N, CA, C, O
Val109	N, CA, C, O, CB, CG1, CG2
Leu110	N, CA, C, O, CB, CG, CD1, CD2
Leu124	N, CA, C, O, CB, CG, CD1, CD2
Val125	N, CA, C, O, CB, CG1, CG2
Asp126	N, CA, C, O, CB, CG, OD1, OD2
Ile127	N, CA, C, O, CB, CG1, CG2, CD1
Asp128	N, CA, C, O, CB, CG, OD1, OD2
Val131	N, CA, C, O, CB, CG1, CG2
Met132	N, CA, C, O, CB, CG, SD, CE

Ser135	N, CA, C, O, CB, OG
Ile142	N, CA, C, O, CB, CG1, CG2, CD1
Gly158	N, CA, C, O
Ile174	N, CA, C, O, CB, CG1, CG2, CD1
Ile175	N, CA, C, O, CB, CG1, CG2, CD1
Asp176	N, CA, C, O, CB, CG, OD1, OD2
Thr177	N, CA, C, O, CB, OG1, CG2
Thr178	N, CA, C, O, CB, OG1, OG2
Phe188	N, CA, C, O, CB, CG, CD1, CD2, CE1, CE2, CZ
Phe192	N, CA, C, O, CB, CG, CD1, CD2, CE1, CE2, CZ
Tyr193	N, CA, C, O, CB, CG, CD1, CD2, CE1, CE2, CZ, OH
Cys207	N, CA, C, O, CB, SG
Asn208	N, CA, C, O, CB, CG, OD1, ND2
Gln209	N, CA, C, O, CB, CG, CD, OE1, NE2
Gly210	N, CA, C, O
Glu211	N, CA, C, O, CB, CG, CD, OE1, OE2
Ser212	N, CA, C, O, CB, OG
Met223	N, CA, C, O, CB, CG, SD, CE
Met240	N, CA, C, O, CB, CG, SD, CE
Val242	N, CA, C, O, CB, CG1, CG2
Thr244	N, CA, C, O, CB, OG1, CG2
Tyr245	N, CA, C, O, CB, CG, CD1, CD2, CE1, CE2, CZ, OH
Pro246	N, CA, C, O, CB, CG, CD
Thr252	N, CA, C, O, CB, OG1, CG2
Leu253	N, CA, C, O, CB, CG, CD1, CD2

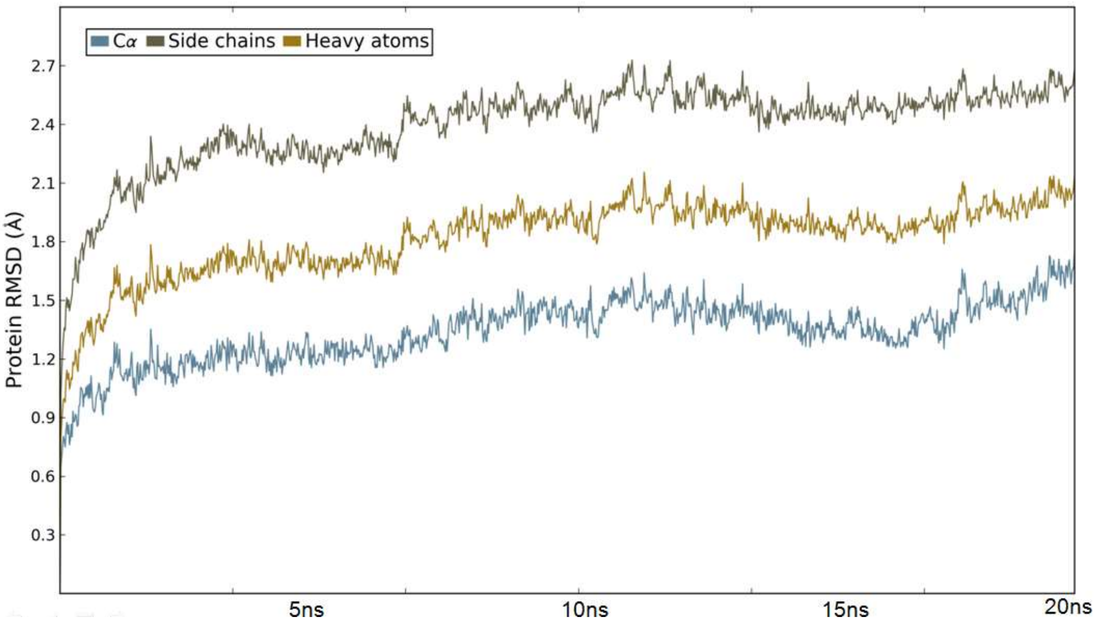

Figure S1. Root-mean-square deviations (RMSD) in the MD simulations. Blue: α-carbon, gray: side chains, yellow: heavy atoms.

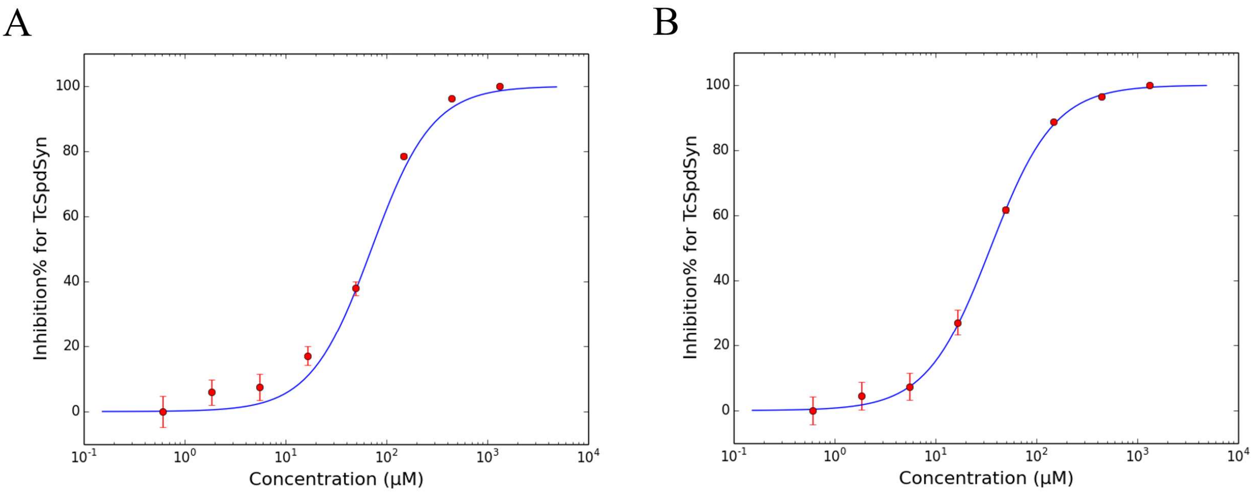

Figure S2. The fitting curves for the calculation of the IC_{50} values in Table 1 (A: compound **1**, B: compound **2**). The error bars represent the standard error of the % inhibition measured at each dose.

Table S2. Crystallography data for the TcSpdSyn–**1** and TcSpdSyn–**2** complexes.

Data collection		Refinement	
Space group	$P2_1$	no. of reflections	57047
a, b, c (Å)	43.8, 99.7, 134.3	R_{work}/R_{free}	0.261/0.292
α, β, γ (deg)	90.0, 91.6, 90.0	no. of atoms (protein)	8649
Resolution (Å)	2.17	no. of atoms (water)	91
R_{merge}	0.077	RMSD	
$I/\sigma I$	16.8	bond lengths (Å)	0.011
Completeness (%)	94.2	bond angles (deg)	1.626
Redundancy	1.7	PDB ID	5Y4P

Data collection		Refinement	
Space group	$P2_1$	No. of reflections	66426
a, b, c (Å)	44.0, 99.7, 134.5	R_{work}/R_{free}	0.224/0.250
α, β, γ (deg)	90.0, 92.0, 90.0	No. of atoms (protein)	8661
Resolution (Å)	2.07	No. of atoms (water)	158
R_{merge}	0.089	RMSD	
$I/\sigma I$	20.2	bond lengths (Å)	0.018
Completeness (%)	98.2	bond angles (deg)	2.131
Redundancy	1.8	PDB ID	5Y4Q

Table S3. SMILES of inactive compounds.

No.	SMILES
3	Cc1[nH]c2c(Cl)cccc2c1CCN
4	Cc1[nH]c2ccc(Cl)cc2c1CCN
5	Cc1[nH]c2ccc([N+](=O)[O-])cc2c1CCN
6	NCc1cc(Cl)c(OCc2ccccc2)c(Cl)c1
7	CC(C(=O)O)N1C(=O)NC2NC(=O)NC21
8	Nc1ccc2c(c1)CC(=O)N2
9	Oc1cccc2c1CCCC2O
10	N#CCc1c[nH]c2ccccc12
11	Oc1ccc2c(O)cccc2c1
12	Nc1cc(C(=O)O)ccc1O
13	NCCc1cccc(F)c1
14	NC1c2ccccc2CC1O
15	O=C(c1cnn(-c2ccccc2)c1)c1cc(F)ccc1O
16	CC(C)(O)c1cn2ccccc2n1
17	OC(c1nc2ccccc2[nH]1)C(O)c1nc2ccccc2[nH]1
18	Cc1cc2nc(O)c(O)nc2cc1C
19	O=c1[nH]c2ccccc2cc1CO
20	CC(O)c1nc2ccccc2[nH]1
21	Cn1ncc(C(N)=O)c1N
22	Nc1ccc(S(N)(=O)=O)cc1N

23 NCCNS(=O)(=O)c1ccccc1
24 NS(=O)(=O)c1ccc2c(c1)NC(=O)CO2
25 Cc1cc2cc(CN)ccc2[nH]1
26 O=C(O)C1=NN(c2nc3ccccc3[nH]2)C(=O)CC1
27 NCc1nnc2ccc(C(F)(F)F)cn12
28 CCn1c(=O)[nH]c2ccccc21
29 NC(=O)c1c(O)cccc1O
30 Oc1ccc2cccc(O)c2n1
31 NC(=O)c1ccccc1C(N)=O
32 OC(CNC1CCCCC1)c1ccccc1
33 Nc1ncnc(O)c1[N+](=O)[O-]
34 COc1ccc2[nH]c(CN)cc2c1
35 Cc1cc(NCCN)c2ccccc2n1
36 NC(=O)C1CCN(CC(O)c2ccccc2)CC1
37 N=C(Nc1cccc(NC(=N)c2cccnc2)n1)c1cccnc1
38 O=c1ccc2ccccc2[nH]1
39 O=C(O)c1ccc2nc(O)c(O)nc2c1
40 Cc1ccc(OCCSc2nc3c(N)ncnc3[nH]2)cc1C
41 O=C(O)COc1ccc(C=Cc2n[nH]c(-c3ccccc3O)n2)cc1
42 Cc1cc(O)nc2nc(O)ccc12
43 OC(CNCCNc1nccc(C(F)(F)F)n1)COc1ccccc1
44 NC(=O)CNC1CC(=O)N(c2ccc(Br)cc2)C1=O
45 N=C1NC2(O)C(=O)c3ccccc3C2(O)N1
46 Cc1cc(C)c2[nH]c(=O)c(=O)[nH]c2c1
47 O=C(CSc1nc2ccccc2[nH]1)Nc1cccc(O)c1
48 Cn1c2c(c(=O)n(C)c1=O)CN(CCN1CCNCC1)CN2
49 NC(=O)c1nn(Cc2cccc(F)c2)cc1[N+](=O)[O-]
50 O=C1NC(Cc2c[nH]c3ccccc23)C(=O)N2CCCC12
51 CC(=O)N1CCC(N)=N1
52 O=C(NN=Cc1ccc(O)cc1O)C1CC1c1ccccc1
53 O=C(NN=Cc1ccc(O)cc1O)C1C2CCCCC21
54 Cn1nc(CN)c2ccccc2c1=O
55 N#CC(=C(O)c1ccccc1)c1nc2ccccc2[nH]1
56 NC(=O)C(Cc1ccccc1)N1CC(=O)NC(=O)C1
57 CC1OC(Nc2cccc([N+](=O)[O-])c2)C(O)C(O)C1O
58 Cc1sc2ncnc(NN=Cc3ccccc3O)c2c1C
59 Nc1nc(-c2ccccc2O)nc(-c2ccccc2O)n1
60 CCN1C(=O)CC(NCCc2ccc(S(N)(=O)=O)cc2)C1=O
61 CC(=O)NC(c1ccccc1)c1c(O)ccc2ccc(O)cc12
62 Cn1c(=O)c2[nH]c(NN)nc2n(C)c1=O
63 NC(=S)Cc1c[nH]c2ccccc12
64 O=C(CSc1nc2cccnc2[nH]1)Nc1ccc(F)cc1
65 Cc1cc(C)nc(NC(=N)N2CCC(C(N)=O)CC2)n1
66 CCN1C(=O)NC2NC(=O)N(CC)C21
67 NC(=O)c1sc2c(c1N)C(c1ccccc1)CC(=O)N2
68 NC(=O)CN1C(=O)C(O)(CC(=O)c2cccc(N)c2)c2ccccc21
69 Cc1ccnc(Nc2nc(-c3ccc(O)cc3O)cs2)c1

70 CC1CC(C)CN(Cc2nc(N)nc(Nc3ccccc3)n2)C1
71 COc1ccc(Nc2nc(-c3ccccc3O)cs2)cc1
72 O=C(CC1Nc2ccccc2NC1=O)NCCc1ccccc1
73 O=C(NCCc1c[nH]c2ccccc12)c1ccccn1
74 Nc1nc(C=Cc2ccccc2)nc(-c2ccccc2O)n1
75 Cc1cccc(NCc2nnc(SCC(N)=O)n2-c2ccccc2)c1
76 CC1=C(C(N)=O)C(c2cccnc2)n2ncnc2N1
77 N=c1oc2ccccc2cc1-c1nc2ccccc2[nH]1
78 NCCNS(=O)(=O)c1ccc(Cl)cc1
79 N=C(N)C1CN(Cc2ccncc2)C(=O)NC1=O
80 CC(C(N)=O)n1cncn1
81 C(=NCCN=Cc1cccs1)c1cccs1
82 Oc1nc2ccccc2nc1F
83 O=C(CN1CCCCC1)Nc1ccc2cn[nH]c2c1
84 CC(=O)N1c2ccccc2NC(=O)C1CC(=O)Nc1cccc([N+](=O)[O-])c1
85 CC(=O)c1cccc(NC(=O)Nc2ccccc2C(N)=O)c1
86 O=C(NN=C1CCN(Cc2ccccc2)CC1)C(O)c1ccccc1
87 Cn1c2c(c(=O)n(C)c1=O)CN(Cc1ccc(F)cc1)CN2
88 CC1(C)OC(C(N)=O)C(C(N)=O)O1
89 OC(CNCC(O)COc1ccccc1)COc1ccccc1
90 NS(=O)(=O)c1cccc(NC(=O)c2cccc(OCc3ccccc3)c2)c1
91 Nc1ncnc2c1ncn2C1OC(CO)C(F)C1O
92 NS(=O)(=O)c1ccc(Cl)c(S(N)(=O)=O)c1
93 OC1CCCN(CC(O)c2ccccc2)C1
94 COc1ccc2nc(C)cc(N3CCC(C(N)=O)CC3)c2c1
95 CC(NCC(O)COc1cccc([N+](=O)[O-])c1)c1ccc(F)cc1
96 COc1ccc(C(=O)C(C#N)c2nc3ccccc3[nH]2)cc1[N+](=O)[O-]
97 CC(=O)c1cccc(OCC(O)CNCC(C)(C)CN(C)C)c1
98 CN1C(=O)c2ccccc2C1O
99 O=C(CC1OC(=O)c2ccccc21)Nc1nc2ccccc2[nH]1
100 N#CC1=C(N)OC2NN=C(C(F)(F)F)C2C1c1ccc(O)cc1
101 CC(=NOCC(=O)NN=Cc1ccc(C)cc1O)c1cccs1
102 Nc1ccccc1Nc1c(N)c(=O)oc2ccccc12
103 Nc1ncnc2c1nc(SCc1ccc3nsnc3c1)n2CC(O)CO
104 O=C(O)CN=Cc1ccc(O)cc1O
105 NC(=NOC(=O)c1ccoc1)c1ccc(Br)cc1
106 Cc1oc(C(N)=O)cc1COc1ccccc1C(N)=O
107 O=C(O)C(Cc1ccccc1)NCC(O)COc1ccccc1
108 O=C(O)C(Cc1c[nH]cn1)NCC(O)COc1ccccc1
109 O=C(O)C1Cc2c([nH]c3ccccc23)C(c2c[nH]c3ccccc23)N1
110 OC(CCNc1ccccc1)c1ccc(Cl)cc1
111 CC1=NN=C(S)SC1C
112 O=c1[nH]c(=O)n(C2CC(O)C(CO)O2)cc1Cl
113 CC(CCc1ccccc1)NCC(O)c1ccccc1
114 Nc1nc2cc3nc(O)c(O)nc3cc2s1
115 Cc1cc(=O)[nH]c(Nc2ccccc2O)n1
116 Cc1cc2[nH]c(=O)c(=O)[nH]c2cc1C

117 Nc1nc2[nH]c(SCc3ccccc3)nc2c(=O)[nH]1
118 O=C(O)C(Cc1c[nH]cn1)NC1CNN=C1c1ccccn1
119 COc1ccc2c(c1O)CN1CCC(=O)CC1C2O
120 CC1(C)OC2C(CO)OC(n3cnc4c(=O)[nH]cnc43)C2O1
121 CC(=O)c1ccc(OCC(O)CN2CCC(C(N)=O)CC2)cc1
122 NC(=O)C1CCN(CC(O)c2cccc([N+](=O)[O-])c2)CC1
123 O=C1NCCCCC1NCC(O)c1ccccc1
124 CC(=O)Cc1c[nH]c2ccccc12
125 Cn1ncc(C(N)=O)c1[N+](=O)[O-]
126 CC(=O)N1CSCC1C(=O)NNS(=O)(=O)c1ccccc1
127 OC(c1nc2cc(F)ccc2[nH]1)C(O)c1nc2cc(F)ccc2[nH]1
128 CC1=C(C(N)=O)C(c2ccc(O)cc2)n2nc(CCCO)nc2N1
129 CC1=C(C(N)=O)C(c2cccc(O)c2)n2nc(CCCO)nc2N1
130 N=c1ccn(CC(N)=O)c2ccccc12
131 NC(=O)CC(NC(=O)OCc1ccccc1)C(=O)OCc1ccccc1
132 O=C1NC2NC(=O)N(CCO)C2N1
133 Cc1ccc2[nH]c3c(c2c1)CCC3C#N
134 CN1C(=O)N(C)C2SC(N)=NNC21
135 CC(N)c1nnc(SCc2ccc(Cl)cc2)o1
136 Nc1nc(C=Cc2ccc(Cl)cc2)nc(-c2ccccc2O)n1
137 CN1CCN(c2nc(O)cc(CSc3nc4ccccc4s3)n2)CC1
138 COc1ccc2[nH]c(=O)c(=O)[nH]c2c1
139 O=C(CC1SC2=NCCN2C1=O)Nc1ccc(Cl)cc1
140 CC(=O)Nc1ccc(OCC(O)CNC2CCCCC2)cc1
141 NS(=O)(=O)c1ccc(CCNc2ncnc3[nH]cnc23)cc1
142 NC(=O)NC(Cc1cccc(OCc2ccccc2)c1)C(=O)O
143 NCCc1c(F)cccc1F
144 CC(C)(C)NCC(O)c1ccc(O)c(CO)c1
145 Cc1nc(O)nc(O)c1C(N)=O
146 Oc1ccc(C=NNc2nc(O)cc(-c3ccccc3)n2)c(O)c1
147 CC1CC(NC(N)=O)NC(=O)N1
148 NC(=O)C1CCCCC1O
149 NC(=S)c1ccccc1O
150 NCC(C(=O)O)c1cccnc1
151 NC(=O)C(N)Cc1ccccc1
152 NC1CCc2ccccc2NC1=O
153 CC(N)c1nc2ccccc2n1C(F)F
154 Nc1nc(CN2CCCCC2)nc(Nc2ccccc2)n1
155 Cc1[nH]nc(O)c1C1OC(=O)c2ccccc21
156 CN(C)c1ccc(CNCCc2c[nH]c3ccccc23)cc1
157 NC(=O)c1cc2ccccc2[nH]1
158 Fc1ccc(CNCC2CCNCC2)cc1
159 [O-][N+](=Cc1ccccc1)Cc1ccccc1
160 NN=C1C(=O)Nc2ccccc21
161 O=C(CCn1ccnc1)Nc1nc2ccccc2[nH]1
162 Cc1ccc(Nc2nc(N)nc(COC(=O)CN3CCCC3=O)n2)cc1
163 O=[N+]([O-])c1c(Nc2ccc3[nH]ncc3c2)nc2sccn12

164 Cn1cc(C(N)=O)c(=O)[nH]c1=O

165 CN(C)C(CN)c1ccc(Cl)cc1

166 Nc1ccn(C2CC(O)C(CO)O2)c(=O)n1

167 CC(N)Cc1cccc(F)c1

168 NCc1ccccc1-c1ccc(Cn2cncn2)cc1

169 Cc1cc(C)n(-c2cc(C(=N)N)ccn2)n1

170 NCc1ccccc1N1CCCC(C(N)=O)C1

171 O=c1[nH]c(Nc2ccccc2O)nc2c1CCCC2

172 NC(=O)NC(CC(=O)Nc1ccc(F)cc1)c1ccccc1

173 NC(CC1C(=O)Nc2ccccc21)C(=O)O

174 NC(=S)c1ccc[nH]1

175 N=C(N)c1ccnc(Oc2cccnc2)c1

176 O=C(O)C1CC(O)c2ccccc21

177 Cc1nc2cc(C3CCCN3C(=O)c3ccccn3)nn2c(O)c1C

178 CCN(Cc1cccc(Cl)c1)CC(O)Cn1cccn1

179 CC(NCC1CNc2ccnn2C1)c1ccc(F)c(Cl)c1

180 CNC(=O)C(N)Cc1c[nH]c2ccccc12

181 CC(C)C(N)c1nnc2n1N=C(c1ccc(Cl)cc1)CS2

182 CC(N)c1nnc(SCc2ccc(Cl)cc2Cl)o1

183 O=C(NCc1ccccc1)NCC(O)c1cccc2ccccc12

184 CN1CCN(c2ccc(C(F)(F)F)cc2CN)CC1

185 FC1(F)CCNCC12CCN(Cc1ccccn1)C2

186 CC(N)Cc1ccc2c(c1)OCC2

187 CC1CCC(C(N)=O)CN1C(=O)c1cn(-c2ccccc2)nn1

188 [O-][n+]1ccc(NO)c2ccccc21

189 OCCN(Cc1ccccc1)CC(O)COCc1ccccc1

190 O=c1[nH]c2ccc(C(F)(F)F)cc2[nH]c1=O

191 Cc1c(CN2CCCC(O)C2)[nH]c2ccc(F)cc12

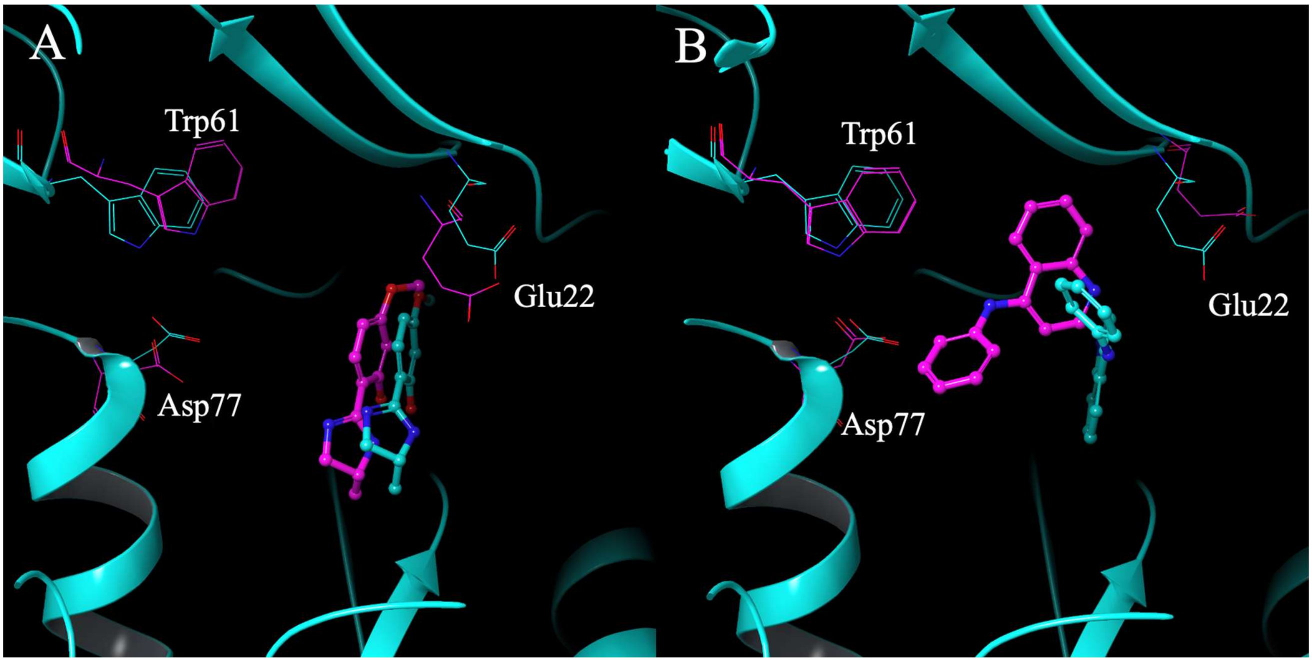

Figure S3. Prediction of structural changes in docking poses by MD simulation. A: Structure of compound **1** at 32 ns., B: Structure of compound **2** at 32 ns. Light blue structure shows the initial docking pose, the purple structure shows the structure after MD simulation.

The additional MD simulations for docking pose of compounds 1 and 2 were conducted using Desmond included in Maestro 2020-3 (Schrödinger, LLC). SpdSyn-compound 1 and 2 complex structures predicted by docking simulations were placed in an orthorhombic box with a buffer distance of 10 Å to create a hydration model, and the SPC water model was used for the hydration model. NaCl (0.15 M) served as the counter ion to neutralize the system. The cutoff radii for van der Waals and the time step, initial temperature, and pressure of the system were set to 9 Å, 2.0 fs, 300 K, and 1.01325 bar, respectively. The sampling interval during the simulation was set to 100 ps. Finally, we performed MD simulations using the NPT ensemble. All of these processes used the OPLS3e force field.
